# Poly[[tris­[μ-2,2′-(butane-1,4-diyl­dithio)bis­(1,3,4-thia­diazole)-κ^2^ *N* ^4^:*N* ^4′^]copper(II)] bis­(perchlorate)]

**DOI:** 10.1107/S1600536809005625

**Published:** 2009-02-21

**Authors:** Pu-Zhou Hu, Jian-Hua Qin, Jian-Ge Wang

**Affiliations:** a College of Chemistry and Chemical Engineering, Luoyang Normal University, Luoyang 471022, People’s Republic of China

## Abstract

In the title compound, {[Cu(C_8_H_10_N_4_S_4_)_3_](ClO_4_)_2_}_*n*_, the Cu^II^ atom is located on a threefold inversion axis coordinated by six N atoms of symmetry-equivalent 2,2′-(butane-1,4-diyl­dithio)bis­(1,3,4-thia­diazole) ligands in a slightly distorted octa­hedral geometry. Adjacent Cu^II^ atoms are linked by the bridging bidentate thia­diazole ligands, which are situated about inversion centers. This leads to the formation of a three-dimensional network structure.

## Related literature

For copper(II) complexes involving the same ligand, see: Huang *et al.* (2009 ▶); Wang *et al.* (2008 ▶).
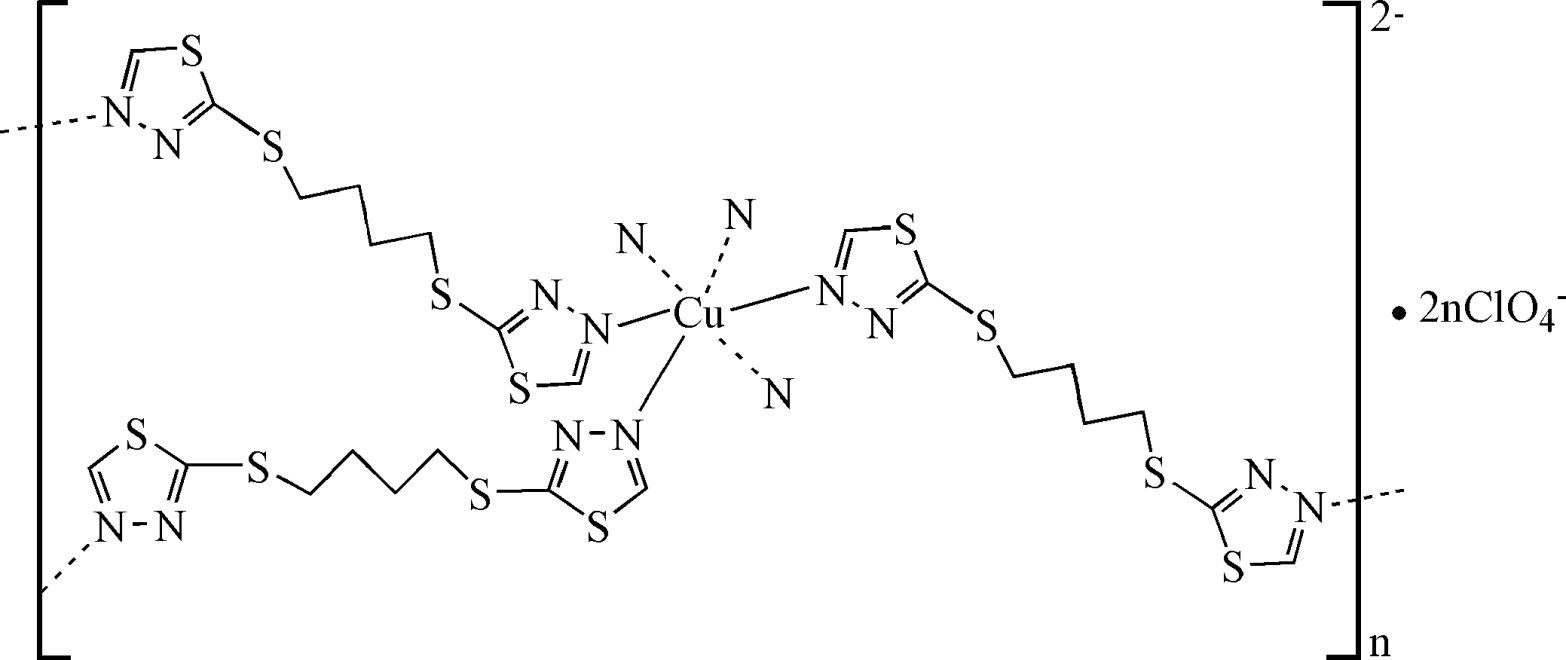


## Experimental

### 

#### Crystal data

[Cu(C_8_H_10_N_4_S_4_)_3_](ClO_4_)_2_
*M* *_r_* = 1133.76
Trigonal, 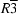

*a* = 10.5455 (6) Å
*c* = 33.728 (4) Å
*V* = 3248.3 (5) Å^3^
*Z* = 3
Mo *K*α radiation
μ = 1.27 mm^−1^
*T* = 291 K
0.28 × 0.21 × 0.14 mm

#### Data collection

Bruker SMART CCD area-detector diffractometer
Absorption correction: multi-scan (*SADABS*; Bruker, 1997 ▶) *T*_min_ = 0.717, *T*_max_ = 0.839
9432 measured reflections
1673 independent reflections
1320 reflections with *I* > 2σ(*I*)
*R*_int_ = 0.028

#### Refinement

*R*[*F* ^2^ > 2σ(*F* ^2^)] = 0.047
*wR*(*F* ^2^) = 0.133
*S* = 1.05
1673 reflections
90 parameters
H-atom parameters constrained
Δρ_max_ = 0.82 e Å^−3^
Δρ_min_ = −0.51 e Å^−3^

### 

Data collection: *SMART* (Bruker, 1997 ▶); cell refinement: *SAINT* (Bruker, 1997 ▶); data reduction: *SAINT*; program(s) used to solve structure: *SHELXS97* (Sheldrick, 2008 ▶); program(s) used to refine structure: *SHELXL97* (Sheldrick, 2008 ▶); molecular graphics: *SHELXTL* (Sheldrick, 2008 ▶); software used to prepare material for publication: *SHELXTL*.

## Supplementary Material

Crystal structure: contains datablocks I, global. DOI: 10.1107/S1600536809005625/su2097sup1.cif

Structure factors: contains datablocks I. DOI: 10.1107/S1600536809005625/su2097Isup2.hkl

Additional supplementary materials:  crystallographic information; 3D view; checkCIF report

## Figures and Tables

**Fig. 1.**
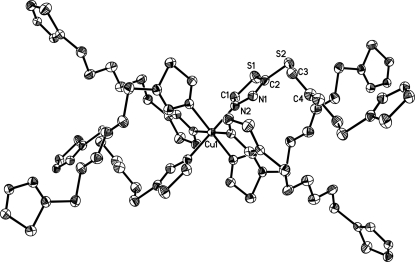
A view of the coordination around the CuII atom in the cation of the title compound. Displacement ellipsoids are drawn at the 30% probability level. The H atoms and perchlorate ion were omitted for clarity.

**Fig. 2.**
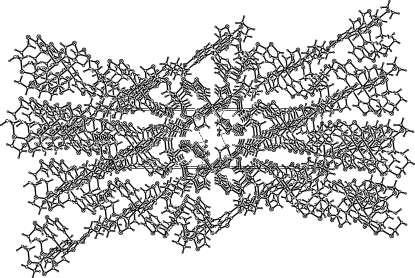
A view down the b axis of the crystal packing of the title compound.

## supplementary crystallographic information

### Comment

The asymmetric unit of the title compound consists of one sixth of a Cu^II^ atom, which is located on a three-fold inversion axis, half a 2,2'-(butane-1,4-diyldithio)bis(1,3,4-thiadiazole) ligand which possesses an inversion center, and one third of a perchlorate ion, which is situated on a three-fold rotation axis. As depicted in Fig. 1, the Cu^II^ atom is coordinated by six N atoms from six symmetry equivalent 2,2'-(butane-1,4-diyldithio)bis(1,3,4-thiadiazole) ligands, in a slightly distorted octahedral geometry of the central atom. The Cu—N bond distance is 2.149 (3) Å, within the range expected for such coordination bonds (Huang *et al.*,  2009; Wang *et al.*,  2008). The centrosymmetric 2,2'-(butane-1,4-diyldithio)bis(1,3,4-thiadiazole) ligand adopts a N,N'-bidentate bridging mode in a trans configuration and links the Cu^II^ atoms to form a three-dimensional network. The bridging Cu···Cu distance is 12.7854 (12) Å (Fig. 2).

### Experimental

The reaction of 2,2'-(butane-1,4-diyldithio)bis(1,3,4-thiadiazole) (0.3 mmol) with Cu(ClO_4_)_2_ (0.1 mmol) in MeOH(10 ml) for a few minutes gave a light blue solid, which was filtered off, washed with acetone, and dried in air. Single crystals, suitable for X-ray analysis, were obtained by slow diffusion of Et_2_O into an acetonitrile solution of the solid.

### Refinement

The H-atoms were positioned geometrically and treated as riding: C—H = 0.93 - 0.97 Å and U_iso_(H) = 1.2U_eq_(parent C-atom).

### Crystal data

**Table:**

[Cu(C_8_H_10_N_4_S_4_)_3_](ClO_4_)_2_	*D*_x_ = 1.739 Mg m^−^^3^
*M**_r_* = 1133.76	Mo *K*α radiation, λ = 0.71073 Å
Trigonal, *R*3	Cell parameters from 2638 reflections
Hall symbol: -R 3	θ = 2.3–24.5°
*a* = 10.5455 (6) Å	µ = 1.27 mm^−^^1^
*c* = 33.728 (4) Å	*T* = 291 K
*V* = 3248.3 (5) Å^3^	Block, blue
*Z* = 3	0.28 × 0.21 × 0.14 mm
*F*(000) = 1731	

### Data collection

**Table:**

Bruker SMART CCD area-detector diffractometer	1673 independent reflections
Radiation source: fine-focus sealed tube	1320 reflections with *I* > 2σ(*I*)
graphite	*R*_int_ = 0.028
φ and ω scans	θ_max_ = 27.5°, θ_min_ = 2.3°
Absorption correction: multi-scan (*SADABS*; Bruker, 1997)	*h* = −13→13
*T*_min_ = 0.717, *T*_max_ = 0.839	*k* = −13→13
9432 measured reflections	*l* = −43→43

### Refinement

**Table:**

Refinement on *F*^2^	Primary atom site location: structure-invariant direct methods
Least-squares matrix: full	Secondary atom site location: difference Fourier map
*R*[*F*^2^ > 2σ(*F*^2^)] = 0.047	Hydrogen site location: inferred from neighbouring sites
*wR*(*F*^2^) = 0.133	H-atom parameters constrained
*S* = 1.05	*w* = 1/[σ^2^(*F*_o_^2^) + (0.0617*P*)^2^ + 10.9603*P*] where *P* = (*F*_o_^2^ + 2*F*_c_^2^)/3
1673 reflections	(Δ/σ)_max_ < 0.001
90 parameters	Δρ_max_ = 0.82 e Å^−^^3^
0 restraints	Δρ_min_ = −0.51 e Å^−^^3^

### Fractional atomic coordinates and isotropic or equivalent isotropic displacement parameters (Å^2^)

**Table:**

	*x*	*y*	*z*	*U*_iso_*/*U*_eq_	
Cu1	1.0000	1.0000	0.5000	0.0338 (2)	
Cl1	0.3333	0.6667	0.45984 (7)	0.0692 (5)	
S1	0.94592 (14)	0.59055 (13)	0.43361 (3)	0.0702 (4)	
S2	0.71904 (11)	0.56831 (12)	0.37580 (3)	0.0608 (3)	
O1	0.3599 (4)	0.8063 (4)	0.47130 (14)	0.1084 (13)	
O2	0.3333	0.6667	0.4165 (2)	0.124 (3)	
N1	0.8519 (3)	0.7722 (3)	0.43379 (8)	0.0500 (7)	
N2	0.9537 (3)	0.8133 (3)	0.46412 (8)	0.0461 (6)	
C1	1.0099 (4)	0.7300 (4)	0.46704 (11)	0.0578 (9)	
H1	1.0802	0.7448	0.4860	0.069*	
C2	0.8366 (4)	0.6570 (4)	0.41560 (10)	0.0501 (8)	
C3	0.6329 (4)	0.6782 (5)	0.36973 (12)	0.0615 (9)	
H3A	0.5323	0.6158	0.3613	0.074*	
H3B	0.6313	0.7208	0.3951	0.074*	
C4	0.7119 (5)	0.8003 (5)	0.33947 (13)	0.0679 (11)	
H4A	0.8032	0.8758	0.3508	0.082*	
H4B	0.7351	0.7613	0.3163	0.082*	

### Atomic displacement parameters (Å^2^)

**Table:**

	*U*^11^	*U*^22^	*U*^33^	*U*^12^	*U*^13^	*U*^23^
Cu1	0.0332 (3)	0.0332 (3)	0.0350 (4)	0.01660 (15)	0.000	0.000
Cl1	0.0537 (6)	0.0537 (6)	0.1003 (14)	0.0269 (3)	0.000	0.000
S1	0.0854 (8)	0.0764 (7)	0.0679 (7)	0.0548 (6)	−0.0153 (5)	−0.0153 (5)
S2	0.0585 (6)	0.0671 (6)	0.0537 (6)	0.0292 (5)	−0.0048 (4)	−0.0096 (4)
O1	0.101 (3)	0.073 (2)	0.152 (4)	0.045 (2)	0.015 (3)	−0.023 (2)
O2	0.140 (4)	0.140 (4)	0.090 (5)	0.070 (2)	0.000	0.000
N1	0.0441 (15)	0.0547 (17)	0.0504 (16)	0.0242 (13)	−0.0014 (12)	−0.0007 (13)
N2	0.0424 (14)	0.0526 (16)	0.0430 (14)	0.0237 (12)	0.0009 (11)	0.0021 (12)
C1	0.063 (2)	0.067 (2)	0.053 (2)	0.040 (2)	−0.0060 (17)	−0.0057 (17)
C2	0.0449 (17)	0.059 (2)	0.0434 (17)	0.0241 (16)	0.0067 (13)	0.0052 (15)
C3	0.051 (2)	0.068 (2)	0.062 (2)	0.0271 (19)	−0.0115 (17)	−0.0019 (19)
C4	0.060 (2)	0.073 (3)	0.075 (3)	0.036 (2)	−0.004 (2)	−0.001 (2)

### Geometric parameters (Å, °)

**Table:**

Cu1—N2^i^	2.149 (3)	S2—C2	1.748 (4)
Cu1—N2	2.149 (3)	S2—C3	1.807 (4)
Cu1—N2^ii^	2.149 (3)	N1—C2	1.298 (4)
Cu1—N2^iii^	2.149 (3)	N1—N2	1.386 (4)
Cu1—N2^iv^	2.149 (3)	N2—C1	1.286 (4)
Cu1—N2^v^	2.149 (3)	C1—H1	0.9300
Cl1—O1	1.409 (4)	C3—C4	1.523 (6)
Cl1—O1^vi^	1.409 (4)	C3—H3A	0.9700
Cl1—O1^vii^	1.409 (4)	C3—H3B	0.9700
Cl1—O2	1.463 (8)	C4—C4^viii^	1.494 (8)
S1—C1	1.702 (4)	C4—H4A	0.9700
S1—C2	1.731 (4)	C4—H4B	0.9700
			
N2^i^—Cu1—N2	91.39 (10)	C2—N1—N2	110.8 (3)
N2^i^—Cu1—N2^ii^	91.40 (10)	C1—N2—N1	113.1 (3)
N2—Cu1—N2^ii^	91.39 (10)	C1—N2—Cu1	127.7 (2)
N2^i^—Cu1—N2^iii^	88.61 (10)	N1—N2—Cu1	119.2 (2)
N2—Cu1—N2^iii^	88.61 (10)	N2—C1—S1	114.9 (3)
N2^ii^—Cu1—N2^iii^	179.998 (1)	N2—C1—H1	122.6
N2^i^—Cu1—N2^iv^	88.61 (10)	S1—C1—H1	122.6
N2—Cu1—N2^iv^	179.999 (2)	N1—C2—S1	114.7 (3)
N2^ii^—Cu1—N2^iv^	88.61 (10)	N1—C2—S2	125.9 (3)
N2^iii^—Cu1—N2^iv^	91.39 (10)	S1—C2—S2	119.4 (2)
N2^i^—Cu1—N2^v^	179.999 (1)	C4—C3—S2	112.3 (3)
N2—Cu1—N2^v^	88.61 (10)	C4—C3—H3A	109.1
N2^ii^—Cu1—N2^v^	88.60 (10)	S2—C3—H3A	109.1
N2^iii^—Cu1—N2^v^	91.39 (10)	C4—C3—H3B	109.1
N2^iv^—Cu1—N2^v^	91.39 (10)	S2—C3—H3B	109.1
O1—Cl1—O1^vi^	112.77 (18)	H3A—C3—H3B	107.9
O1—Cl1—O1^vii^	112.77 (18)	C4^viii^—C4—C3	111.9 (4)
O1^vi^—Cl1—O1^vii^	112.77 (18)	C4^viii^—C4—H4A	109.2
O1—Cl1—O2	105.9 (2)	C3—C4—H4A	109.2
O1^vi^—Cl1—O2	105.9 (2)	C4^viii^—C4—H4B	109.2
O1^vii^—Cl1—O2	105.9 (2)	C3—C4—H4B	109.2
C1—S1—C2	86.55 (18)	H4A—C4—H4B	107.9
C2—S2—C3	101.18 (18)		
			
C2—N1—N2—C1	0.6 (4)	Cu1—N2—C1—S1	179.51 (16)
C2—N1—N2—Cu1	−179.3 (2)	C2—S1—C1—N2	0.1 (3)
N2^i^—Cu1—N2—C1	84.4 (4)	N2—N1—C2—S1	−0.6 (4)
N2^ii^—Cu1—N2—C1	175.8 (3)	N2—N1—C2—S2	179.7 (2)
N2^iii^—Cu1—N2—C1	−4.2 (3)	C1—S1—C2—N1	0.3 (3)
N2^v^—Cu1—N2—C1	−95.6 (4)	C1—S1—C2—S2	−179.9 (2)
N2^i^—Cu1—N2—N1	−95.76 (17)	C3—S2—C2—N1	−0.8 (4)
N2^ii^—Cu1—N2—N1	−4.3 (2)	C3—S2—C2—S1	179.4 (2)
N2^iii^—Cu1—N2—N1	175.7 (2)	C2—S2—C3—C4	93.0 (3)
N2^v^—Cu1—N2—N1	84.24 (17)	S2—C3—C4—C4^viii^	165.9 (4)
N1—N2—C1—S1	−0.4 (4)		

Symmetry codes: (i) −*y*+2, *x*−*y*+1, *z*; (ii) −*x*+*y*+1, −*x*+2, *z*; (iii) *x*−*y*+1, *x*, −*z*+1; (iv) −*x*+2, −*y*+2, −*z*+1; (v) *y*, −*x*+*y*+1, −*z*+1; (vi) −*x*+*y*, −*x*+1, *z*; (vii) −*y*+1, *x*−*y*+1, *z*; (viii) −*x*+4/3, −*y*+5/3, −*z*+2/3.
